# Deleted in Malignant Brain Tumors-1 Protein (DMBT1): A Pattern Recognition Receptor with Multiple Binding Sites

**DOI:** 10.3390/ijms1112521

**Published:** 2010-12-17

**Authors:** Antoon J. M. Ligtenberg, Niclas G. Karlsson, Enno C. I. Veerman

**Affiliations:** 1 Periodontology and Oral Biochemistry, Academic Centre for Dentistry Amsterdam, G. Mahlerlaan 3004, 1081LA, Amsterdam, The Netherlands; E-Mail: e.veerman@acta.nl; 2 Medical Biochemistry, University of Gothenburg, Gothenburg, Sweden Box 440, 40530 Gothenburg, Sweden; E-Mail: niclas.karlsson@medkem.gu.se

**Keywords:** dental caries, innate immunity, mucosal protection, SRCR domains

## Abstract

Deleted in Malignant Brain Tumors-1 protein (DMBT1), salivary agglutinin (DMBT1^SAG^), and lung glycoprotein-340 (DMBT1^GP340^) are three names for glycoproteins encoded by the same *DMBT1* gene. All these proteins belong to the scavenger receptor cysteine-rich (SRCR) superfamily of proteins: a superfamily of secreted or membrane-bound proteins with SRCR domains that are highly conserved down to sponges, the most ancient metazoa. In addition to SRCR domains, all DMBT1s contain two CUB domains and one zona pellucida domain. The SRCR domains play a role in the function of DMBT1s, which is the binding of a broad range of pathogens including cariogenic streptococci, *Helicobacter pylori* and HIV. Mucosal defense proteins like IgA, surfactant proteins and lactoferrin also bind to DMBT1s through their SRCR domains. The binding motif on the SRCR domains comprises an 11-mer peptide in which a few amino acids are essential for binding (GRVEVLYRGSW). Adjacent to each individual SRCR domain are glycosylation domains, where the attached carbohydrate chains play a role in the binding of influenza A virus and *Helicobacter pylori*. The composition of the carbohydrate chains is not only donor specific, but also varies between different organs. These data demonstrate a role for DMBT1s as pattern recognition molecules containing various peptide and carbohydrate binding motifs.

## 1. Introduction: Saliva as a Protective Fluid

Mucosal surfaces are the largest and most important interface between the human body and its environment, comprising a total area of approximately 300 m^2^ [1]. Interaction with the environment is of vital importance for the uptake of nutrients and oxygen, but the external interaction of mucosal surfaces also poses a threat the body. These threats include antigenic, mitogenic and toxic stimuli in food and in the air as well as pathogenic bacteria and viruses. To overcome these constant challenges, the immune system has developed extensive innate and adaptive responses.

The oral tissues, a part of the mucosal immune system, are constantly covered by saliva, which harbors a similar set of antimicrobial proteins as other mucosal fluids [2]. A major role of saliva is to maintain the natural oral microbial ecosystem. The function and secretion of saliva can be disturbed after radiation therapy for head and neck cancer, by auto-immune diseases affecting glandular tissues such as Sjögren’s syndrome [3,4] or as a side-effect of numerous drugs. A reduced saliva secretion leads to a significant increase in oral bacteria as well as to a shift in composition of the oral microflora. At the same time, the oral complications increase in number and severity [5]. Reduced saliva secretion may also affect general health. For instance, the majority of sedated patients in intensive-care units show a shift in the oral microflora from Gram-positive to Gram-negative species, which subsequently may spread into the respiratory tract causing pulmonary afflictions [6].

## 2. Adherence and Oral Infections

As a result of the continuous flow of saliva in our mouth, planktonic bacteria are swallowed, making microbial adherence one of the key selective criteria in the oral cavity [7,8]. Salivary proteins in solution may inhibit adherence by competition with bacterial binding sites on proteins coated at dental surfaces. Binding of salivary proteins to bacteria may result in bacterial agglutination which is, therefore, considered as a mechanism for clearance of bacteria and protection against dental caries [9–11]. One of the important bacteria agglutinating proteins is Deleted in Malignant Brain Tumors-1 protein (DMBT1) also known as Salivary Agglutinin (DMBT1^SAG^) [12,13].

## 3. Deleted in Malignant Brain Tumors-1 (DMBT1), Salivary Agglutinin (DMBT1^SAG^) and Glycoprotein 340 (DMBT1^GP340^)

*Streptococcus mutans* is considered the major cause for dental caries. In search for *S. mutans* agglutinating substances, Ericson and Rundegren isolated a 300–400 kDa glycoprotein from parotid saliva [12] that showed calcium-dependent binding to *S. mutans.* This protein was named salivary agglutinin (DMBT1^SAG^). It also binds a number of both Gram-positive and Gram-negative bacteria, as well as to host proteins including IgA, complement factor C1q, bovine and human lactoferrin, albumin and lysozyme [14–19]. Several studies have shown that the protein core of DMBT1^SAG^ is identical with lung glycoprotein 340 (DMBT1^GP340^) and Deleted in Malignant Brain Tumors-1 (DMBT1) [20–22]. The amino acid sequences characterized so far are identical to the one deduced from the *DMBT1* gene [23,24]. DMBT1^SAG^ and DMBT1^gp-340^ cross react with monoclonal antibodies raised against the DMBT1s isolated from the different sources. Both proteins show calcium-dependent binding to *S. mutans* and SP-D [21]. DMBT1^GP340^ was purified from bronchoalveolar lavage fluid of patients with alveolar proteinosis [20]. It binds to surfactant proteins A (SP-A) and D (SP-D) [25], members of the collectin family that play an important role in innate immunity by binding to specific carbohydrate structures on the surface of pathogenic micro-organisms [26]. DMBT1^SAG^ and DMBT1^GP340^ thus represent DMBT1 isoforms that are encoded by the *DMBT1* gene on chromosome 10q26.13. The *DMBT1* gene shows frequent genomic rearrangements and/or loss of expression in two common types of malignant brain tumors, but also in diverse epithelial cancer types, including lung, gastric, esophageal, colon, breast, and skin cancer [27–36]. A search through the human genome revealed only one copy of the gene. Genetic polymorphism results in variants with different numbers of SRCR exons. In addition, different isoforms may arise through alternative splicing or differential post-translational modifications such as glycosylation.

## 4. The Domain Organization of DMBT1

**Figure 1.**
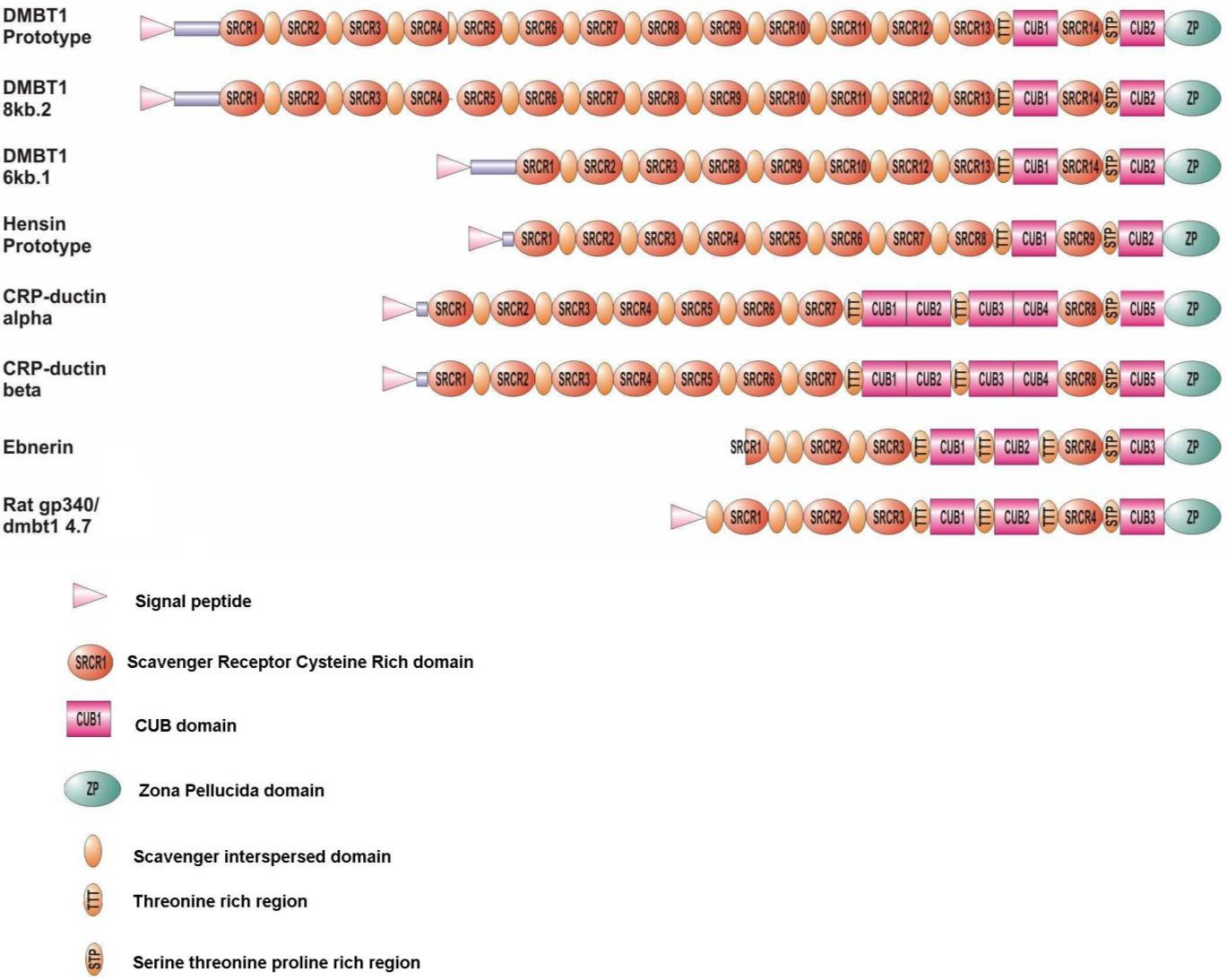
Domain organization of DMBT1 and DMBT1 orthologs. This picture was adapted from J.Mollenhauer [35]. DMBT1/8 kb.2 and DMBT1/6 kb.1 represent the largest and the smallest human variant, respectively, that have been recovered so far. The DMBT1 prototype features 13 scavenger receptor cysteine rich (SRCR) domains, separated by SIDs. The SRCR domains are followed by a short Thr-rich region, a CUB domain, a 14th SRCR domain, a Ser-Thr-Pro-rich region, a second CUB domain, and a Zona Pellucida domain. The DMBT1 orthologs from the rabbit (Hensin), Mouse (CRP-ductin-alfa), rat (Ebnerin) and pig have varying numbers of SRCR domains, followed by one or more CUB domains, and share a *C*-terminal SRCR domain, CUB domains and Zona Pellucida domain.

The most typical feature of DMBT1 is that it consists of protein domains (Figure 1). DMBT1 is a member of the scavenger receptor cysteine-rich (SRCR) superfamily, which is characterized by the presence of one or more SRCR domains [23,24]. In between each of the multiple SRCR domains, all located to the *N*-terminal end of the DMBT1s, are short serine-threonine-rich amino acid motifs of 20–24 amino acids in length, called SRCR interspersed domains (SIDs). The largest naturally occurring allele known to date contains 13 of these SRCR-SID units, while the shortest known displays only eight. This genetic variability in normal individuals raises the question if size variations of DMBT1^GP340^, DMBT1^SAG^ and variants from other tissues are due to alternative splicing and post-translational processing or are alternatively determined by genetic differences.

The stretch of tandem repeated SRCR domains with their interspersed SIDs are followed by two CUB domains separated by a 14th SRCR domain, and at the *C*-terminal end of the gene is a Zona Pellucida domain. Except for the SIDs, these domains are all known to be involved in ligand binding [37–39]. DMBT1 orthologs have been identified in various mammalian organisms such as mouse (dmbt1, CRP-ductin, vomeroglandin, muclin, apactin), rat (dmbt1, ebnerin, pancrin), rabbit (hensin), cow (bovine gall bladder mucin), pig (dmbt1) and rhesus monkey (H3) (Table 1). These proteins all have in common that they consist of SRCR, CUB and ZP domains although the number and order of these domains may be different (Figure 1).

## 5. SRCR Domains

The most prevalent domains in DMBT1 are the SRCR domains [38]. The SRCR superfamily consists of both membrane bound and secreted proteins which play a role in ligand binding [40]. SRCR proteins are present in multicellular animals along the entire animal kingdom, with their earliest appearance in sponges [38,41,42]. In addition, they also appear in protozoan parasites such as *Cryptosporidium*, *Toxoplasma* and *Plasmodium* and algae of the genus *Chlamidomonas* [43,44]. The organism with the highest number of SRCR domains is the purple sea urchin, *Strongylocentrotus purpuratus*, which contains 218 genes comprising altogether 1,095 SRCR domains [45]. For these SRCR proteins, a role as innate immune receptors was suggested since this organism also has a high number of innate immune receptors such as Toll-like receptors. Despite their presence in a large variety of animals [42], SRCR domains have retained a high degree of conservation. SRCR domains are ∼100–110 amino acids long and can be subdivided into two groups: members of group A contain SRCR domains with six cysteine residues, which are encoded by two exons; members of group B, to which DMBT1 belongs, usually contain SRCR domains with eight cysteine residues and are encoded by a single exon [38]. The position of the cysteines and their disulfide bond pattern are well conserved within each SRCR domain. The disulfide bond pattern of a group B SRCR domain is C1–C4, C2–C7, C3–C8 and C5–C6 [42].

## 6. CUB Domains

The CUB domain is a 100–110 residue-spanning extracellular domain named after the three proteins, in which it was first recognized: **C**omplement subcomponents (C1s/C1r), embryonic sea urchin protein (**U**egf; sea urchin epidermal growth factor), and bone morphogenetic protein 1 (**B**mp1). CUB domains are found in numerous proteins that are mainly involved in developmental processes [39]. Almost all CUB domains contain four conserved cysteines, which probably form two disulfide bridges (C1–C2, C3–C4). The structure of the CUB domain has been predicted to be a beta-barrel similar to that of immunoglobulins [46].

## 7. Zona Pellucida Domains

The zona pellucida (ZP) domain was first recognized in the sperm receptor proteins ZP2 and ZP3 [39,47]. These proteins are, along with ZP1, responsible for sperm adhesion to the zona pellucida, the glycoprotein membrane surrounding the plasma membrane of the oocyte. The ZP domain is a ∼260 amino acid module which contains eight conserved cysteines, forming four disulfide bridges. The disulfide bonding pattern suggests that the ZP domain consists of two subdomains. In addition to the conserved cysteines, a few aromatic or hydrophobic amino acids are absolutely invariant [48]. ZP domains are usually present in glycosylated proteins containing other domains and usually located at the *C*-terminal of the polypeptide, which is also the case for DMBT1s. The functions of proteins containing a ZP domain vary tremendously. They are found in glycoproteins involved in development, acoustic perception, immunity, and cancer [49]. ZP domains have been suggested to play a role in protein oligomerization [50]. This is consistent with the model that DMBT1^SAG^ forms aggregates [51,52].

## 8. Glycosylation Patterns of DMBT1^SAG^

The carbohydrate part of DMBT1^SAG^ accounts for 25–40% of the molecular weight [23,52]. DMBT1s contains up to 14 potential *N*-linked glycosylation sites [23], and numerous potential *O*-linked sites situated primarily in the SIDs. The SRCR domains, however, contain only few potential *O*-glycosylation sites (Ser and Thr residues). Presumably, the high density of glycans forces the SIDs in an extended conformation, as has been demonstrated for mucins [53], thereby creating a molecule with alternating stretched SIDs and globular SRCR domains.

The dominating *O*-linked structures in DMBT1^SAG^ are extended core 1 and core 2 oligosaccharides, either neutral or monosialylated (Table 2). These structures are extended by fucosylated oligo-*N*-acetyllactosamine units. Sialylation and fucosylation are found terminating the *N*-acetyllactosamine chains as Sialyl-Le^x^ or Le^b^/Le^y^, respectively. Sialylated structures tend to be shorter than fucosylated structures, suggesting that sialylation regulates the *N*-acetyllactosamine extension.

Glycosylation of proteins is controlled by genetically encoded glycosyltransferases [54]. The Se gene, which determines the presence of blood group antigens in secretory fluids, encodes a α1,2-fucosyltransferase. This enzyme couples a fucose to galactose (Fucα1-2Gal), which is the first step in the generation of blood group antigens. Consequently, non-secretors have a different carbohydrate composition compared to secretors. Non-secretors have Le^a^ and Le^x^ structures on DMBT^SAG^; secretors also have Le^b^ and Le^y^ and ABH structures, in addition to Le^a^ and Le^x,^ on DMBT1^SAG^ [13,55]. DMBT1^SAG^ from secretors has a higher molecular mass than DMBT1^SAG^ from non-secretors [55]. Although evidence has been obtained that blood group antigens such as ABH and the Le^a^ antigens function as ligands for bacterial receptors, and thus might be involved in bacterial binding [56–58], only a few papers describe a correlation between blood group status and the susceptibility to caries [59].

Glycosylation of lung DMBT1^GP340^ is different from that of salivary DMBT1^SAG^. Lung DMBT1^GP340^ completely lacks ABH and Le antigens [55] and its content of α2,3 linked sialic acid residues seems to be lower than that of salivary DMBT1^SAG^ [60]. It is not clear, however, to what extent this difference is related to individual variation in glycosylation, as the preparations were from different donors. Differences are also found for tear DMBT1 when compared to saliva DMBT1^SAG^. In contrast to saliva, DMBT1 in tears had no sialyl-Le^x^. [61].

Also, time-dependent variations in glycosylation patterns have been reported. The reactivity of DMBT1^SAG^ with antibody MECA-79, which recognizes the L-selectin ligand SO_3_-6GlcNAc [62,63], fluctuates during the ovulation cycle. It is low shortly after menstruation and reaches a maximum a few days after ovulation. Also, during pregnancy and lactation, reactivity is high. These results suggest that sulfation of DMBT1^SAG^ is hormonally regulated [63].

## 9. Bacteria Binding Sites on DMBT1^SAG^

### 9.1. Binding Sites on the SRCR Domains

**Figure 2.**
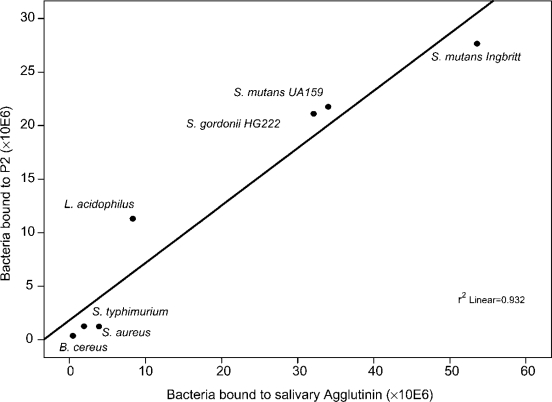
Correlation between bacteria binding to DMBT1^SAG^ and SRCRP2 [67].

For DMBT1 ^SAG^, the SRCR domains are identified as bacteria-binding sites. Proteolytic cleavage of DMBT1^SAG^ with Lys-C results in a protein fragment of 1722 amino acids, only containing SRCR domains and SIDs, which still binds to *S. mutans* [65]. The SRCR domains in this protein fragment show a high degree of homology, which justifies the design of a consensus sequence. Based on this consensus sequence, synthetic peptides were designed consisting of the fragments between the cysteine residues covering the full SRCR domain and SID fragments. Bacterial binding was restricted to a 16-mer peptide (QGRVEVLYRGSWGTVC) representing the second fragment in the SRCR domain and therefore named SRCRP2 [65]. This peptide binds *S. mutans* and a number of other bacterial species including *Streptococcus gordonii*, *Staphylococcus aureus*, *Escherichia coli* and *H. pylori* [66]. Bacterial adhesion to the peptide correlates with adhesion to the full molecule (Figure 2) [67].

*N*- and *C-*terminal truncation resulted in an 11-mer peptide still able to bind bacteria, therefore named DMBT1 pathogen binding site 1 (DMBT1pbs1) [66]. Although bacterial binding within the SRCR domains is only restricted to the 11-mer peptide, within the peptide different residues are essential for binding of different bacteria. Alanine scanning analysis demonstrated that eight residues out of 11 were involved in binding of different bacterial species (GRVEVLYRGSW). Of these eight residues, four were always present in the binding motif (GRVEVLYRGSW). Of the 14 SRCR domains in the full length molecule, 12 contain the eight amino acids involved in binding giving bacterial binding of DMBT1 a multivalent character. One of the two deviating SRCR domains is the 14th SRCR domain (GRVEIYHGGTW), located between two CUB domains and as such probably not involved in bacterial binding.

SRCR domains in other proteins have previously been implicated in bacteria binding [68–70]. The bacteria binding site locates in a similar region of the group A SRCR domains in MARCO, which is a macrophage receptor recognizing bacterial surface components such as LPS and LTA. In this case, an RXR-motif, adjacent to the SRCRP2 peptide motif, is responsible for bacterial interactions (GSSN***RGR***AEVYYSG) [71,72]. Similarly, the bacteria binding sites in human Spα and CD6, both group B members of the SRCR superfamily that are expressed by lymphoid macrophages and lymphocytes, are also located in the SRCR domain. [73,74]. CD 163 (M130), a macrophage receptor only consisting of nine SRCR domains, also binds bacteria. Peptides homologous to the DMBT1^SAG^ bacteria binding site were synthesized for all nine SRCR domains and tested for bacteria binding [75]. One of these nine peptides, of SRCR domain 3, showed good binding to bacteria (GRIEIKFQGRW).

### 9.2. Binding Sites on the Glycan Chains

Studies of bacterial binding sites on SAG were first mainly focused on carbohydrate ligands, following the concept that bacteria primarily recognize carbohydrate ligands on mucosal surfaces [76,77]. These studies revealed that DMBT1^SAG^-mediated agglutination of *S.mutans* is inhibited by high concentrations of fucose and lactose. In line with these results, it was shown that the Le^a^-antigen (Galβ1, 3(Fucα1, 4)GlcNAc) was involved in *S.mutans* binding, harboring an essential role for the terminal fucose [13]. Other studies demonstrated that sialic acid plays a role in binding of DMBT1^SAG^ to *S. sanguis* and *S. mutans* [52,78]. *H. pylori* can bind Le^b^ structures and sialic acid on DMBT1^SAG^ with its BabA and SabA adhesins, respectively [64].

## 10. DMBT1^SAG^ Binding Sites on Bacteria

### 10.1. Antigen I/II Polypeptides

DMBT1^SAG^ was first described as an agglutinating agent for *S.mutans* and other streptococci [12]. Several studies have underlined the importance of bacterial agglutination in the protection against dental caries [9,79–81]. High concentrations of DMBT1^SAG^ on the dental surface promoted *S. mutans* colonization, but high concentrations in saliva inhibit its colonization [80].

**Figure 3.**
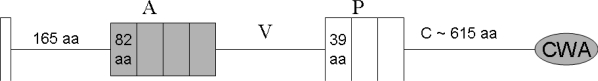
Schematic overview of the structural organization of antigen I/II polypeptides. All proteins contain a 38 residues leader peptide. 165 amino acid residues downstream of the leader peptide is the alanine-rich region containing 1–4 repeats of an 82 residues alanine-rich repeat. This is followed by a variable (V) region, followed by proline-rich P region containing 1–3 copies of a 39 residues repeat. The *C*-terminus shows ≥64% homology along the antigen I/II protein family. *C*-terminally is the cell wall anchoring sequence (CWA).

DMBT1^SAG^ binds in a calcium-dependent manner to antigen I/II polypeptides, a group of surface receptors on *S. mutans* and related streptococci. Antigen I/II polypeptides have been characterized under different names in a variety of streptococci, *S. mutans* (antigen B, P1, Pac, SpaP, MSL-1), *S. sobrinus* (PAg, SpaA), *S. gordonii* (SspA, SspB), *S. intermedius* (Pas), *S. pyogenes* (aspA) and *S. agalactiae* (bspD) [82,83]. Antigen I/II polypeptides on oral streptococci have been studied extensively as candidates for vaccine development [84]. These polypeptides, between 826 and 1653 amino acids long, share a common primary sequence (Figure 3). All proteins start with a 38 amino acid (aa) leader sequence that is cleaved off during secretion. The A-region, containing one to four copies of an 82 aa residues alanine-rich sequence, is found 165 aa residues downstream of this leader. This is followed by a variable region (V-region) and then by a proline-rich P-region containing one to three copies of a 39 residue sequence block. The *C*-terminal region of about 615 amino acids shows 64% or more sequence homology between the I/II polypeptides.

A number of putative binding sites for DMBT1^SAG^ were identified on antigen I/II polypeptides and other proteins (Table 3). Insight has come from the elucidation of the tertiary structure of antigen I/II [83,93]. The V-region of Ag I/II polypeptides may adopt a lectin-like conformation, supporting the suggestion that antigen I/II polypeptides bind to DMBT1^SAG^ by recognition of carbohydrate moieties [94]. This V-region is projected from the cell surface by a stalk formed by an association between an *N*-terminal α-helix of the alanine-rich repeats and a *C*-terminal polyproline helix. The *C*-terminus of antigen I/II, which is in close vicinity to the cell surface, also binds DMBT1^SAG^ [85,93]. A synthetic 20-mer peptide, corresponding to residues 1025-1044 of antigen I/II (PQLKTADLPAGRDETTSFVLV), inhibited binding to DMBT1^SAG^ and selectively inhibited colonization of *S.mutans* to the tooth surface [85]. The presence of two binding sites for DMBT1^SAG^ may also explain the intriguing observation that streptococcal binding to surface immobilized DMBT1^SAG^ is different from binding to DMBT1^SAG^ in solution [95,96]. Overlapping, but not identical, subsets of monoclonal antibodies against antigen P1 inhibited SAG mediated adherence and aggregation, indicating that in the adsorbed state, other domains were involved in the interaction with bacteria than in the soluble phase. Furthermore, DMBT1^SAG^ in solution and adsorbed DMBT1^SAG^ were differentially recognized by streptococci, which formed three phenotypic groupings according to their modes of interaction: one group binding to both surface-bound and soluble DMBT1^SAG^, one group binding only to surface-bound SAG, and one group interacting preferentially with soluble DMBT1^SAG^ [95]. Deletion of antigen I/II polypeptides in *S. gordonii* reduced adhesion to immobilized DMBT1^SAG^ by 40%, while deletion of Hsa—another streptococcal surface antigen—reduced adhesion by 80%. In contrast, aggregation by DMBT1^SAG^ disappeared after deletion of antigen I/II polypeptides but was not affected by deletion of Hsa [97]. The differences in binding between absorbed and soluble state is not unique to DMBT1^SAG^, and has been reported for the protein-bacterial interaction between *Actinomyces viscosus* and acidic proline rich proteins as well as statherin. *A. viscosus* only bound to surfaces coated with these proteins, but not to the same proteins in solution. [98,99]. This is attributed to a conformational change which takes place when the proteins are adsorbed onto a surface, resulting in the exposure of previously hidden bacterial receptors, cryptitopes. Statherin is a peptide which in solution has no defined tertiary structure. Upon adsorption onto a hydroxyapatite surface, the *C*-terminal region folds into a α-helical conformation, which is recognized by bacterial receptors [100]. Conclusive evidence for differences in soluble and surface bound DMBT1^SAG^ remains to be demonstrated by identifying the epitopes that are exposed only on immobilized DMBT1^SAG^ compared to soluble DMBT1^SAG^, and *vice versa*.

### 10.2. Pathogen Associated Molecular Patterns

Bacteria that do not possess antigen I/II polypeptides, like *H. pylori* and *Staphylococcus aureus*, also bind DMBT1^SAG^ [22,65]. CRP-ductin, the mouse homolog of DMBT1, bind to several Gram^+^ and Gram^−^ bacteria, including *Haemophilus influenzae*, *Klebsiella oxytoca*, *S. aureus* and *Streptococcus pneumoniae* [101]. As many SRCR proteins are pattern recognition receptors, DMBT1^SAG^ might also recognize common motifs on pathogens, so–called PAMPs (pathogen associated molecular patterns). DMBT1^SAG^ binds to lipoteichoic acid, a component on the membrane of Gram-positive bacteria, and to lipopolysaccharide (LPS), a common component on the cell membrane of Gram-negative bacteria. LPS has been found to bind to SRCRP2, suggesting DMBT1 recognizes LPS with its SRCR domains [102]. Binding studies to truncated variants of LPS from Salmonella showed that binding depended on the availability and accessibility of phosphorylated structures on LPS. In addition, sulfated substances such as dextran sodium sulfate (DSS) and the food stabilizer carrageenan also bound to DMBT1. DSS is used to induce colitis in mice in a disease model for ulcerative colitis. In a DSS-induced colitis model, *Dmbt1*^−/−^ mice were more susceptible to low doses of DSS than *Dmbt1*^+/+^ mice. However, since this is a commensal bacteria induced colitis model, this effect is likely to be attributed to Dmbt1’s ability to control the natural intestinal microflora.

## 11. Interaction with Viruses

Besides bacteria, DMBT1^SAG^ also binds viruses, including HIV-1 [92,103] and influenza A virus [60,104]. HIV-1 infects host cells by binding to the CD4 receptor through the viral envelope glycoprotein gp120, which leads to conformational changes in gp120 allowing high affinity interaction with chemokine receptors [105,106]. DMBT1^SAG^ shows a calcium-dependent interaction with the virus gp120. The DMBT1^SAG^ binding region on gp120 is localized to a linear, highly conserved sequence (CTRPNYNKRKR) near the stem of the V3 loop that is critical for chemokine receptor interaction during viral binding and infection [92]. Thus, DMBT1^SAG^ most probably blocks the access of gp120 to the chemokine receptor. The *N*-terminal sequence of DMBT1, including the first SRCR domain and one-half of the first SID, binds in a Ca^2+^-dependent manner to the same HIV-1 V3 sequences previously identified to interact with full-length SAG [107].

DMBT1^SAG^ and DMBT1^GP340^ also inhibit the hemagglutination activity and infectivity of Influenza A virus (IAV) [104], which is responsible for outbreaks of influenza. IAV infects cells of the respiratory tract by binding to sialic acid residues present at the cell surface. Different influenza strains recognize specifically α(2,3)-linked or α(2,6)-linked sialic acid. DMBT1 has broad antiviral activity against human, equine and porcine IAV strains by sialic acid mediated binding to IAV, which results in virus neutralization [60]. A variant of DMBT1^SAG^ had significantly greater inhibitory activity against avian-like IAV strains which preferentially bound sialic acids in α(2,3) linkage, than DMBT1^SAG^ from another donor or three lung preparations of DMBT1^GP340^ [60]. In line with this, a greater density of α(2,3)-linked sialic acids was present on the DMBT1^SAG^ from this donor as compared with the other samples. Hence, the specificity of sialic acid linkages on DMBT1 is an important determinant of anti-IAV activity. In addition to these direct virus-neutralizing properties, DMBT1^GP340^ displays cooperative interactions with SP-D in viral neutralization and aggregation assays. On the other hand, in some cases DMBT1^SAG^ strongly antagonizes the antiviral activities of SP-D by binding to its carbohydrate recognition domain, which is involved in virus binding [60].

## 12. Interaction with Endogenous Protein Ligands

DMBT1 binds to a number of endogenous proteins such as secretory IgA, MUC5B, surfactant proteins A and D, complement factor C1q, lactoferrin and albumin [16–18,21,25,89,108,109]. DMBT1^SAG^ binds to secretory IgA in a calcium-dependent manner, resulting in a cooperative effect on bacterial aggregation [15,110]. Binding of DMBT1^SAG^ to IgA is mediated by the same 11-mer peptide that is responsible for the broad-spectrum bacteria binding of DMBT1^SAG^ [18]. Binding of a DMBT1^SAG^-IgA complex to the Pac molecule of *S. mutans* was demonstrated [52]. After chemical reduction, this complex was dissociated into sIgA and DMBT1^SAG^ and concomitantly binding to Pac disappeared.

DMBT1^SAG^ also binds to C1q of the complement system, a system of plasma proteins that may react with one another to opsonize or permeabilize pathogens and induce a series of inflammatory responses [109]. By binding of SAG to C1q, the complement system is activated through the classical pathway [16]. Although DMBT1^SAG^ is secreted in saliva and C1q is a serum component, these two fluids may mix in the oral cavity under conditions of oral inflammation, e.g., periodontal disease, or mucosal damage, thus enabling a local complement activation.

DMBT1 also binds to bovine and human lactoferrin, an 80 kDa iron binding protein belonging to the transferrin family [17,19]. Lactoferrin exhibits various functions in the innate immune system. It sequesters iron from the local environment thus inhibiting microbial growth and it prevents the formation of *Pseudomonas* biofilms [111]. In addition, antimicrobial peptides are released upon proteolytic degradation of lactoferrin [112]. Bovine lactoferrin inhibits DMBT1^SAG^ binding to *S. mutans* protein antigen Pac, which belongs to the antigen I/II family [17]. The SRCRP2 peptide of DMBT1^SAG^ that is responsible for *S. mutans* binding also mediates binding of lactoferrin [17,89]. Bovine lactoferrin residues 480–492 (SCAFDEFFSQSCA) are important for DMBT1^SAG^ binding. Although the homologous sequence in human lactoferrin is slightly different (SC**K**FDE**Y**FSQSCA), human lactoferrin also binds to DMBT1 [19].

DMBT1^GP340^, which originally was isolated as a soluble receptor for SP-D, also binds SP-A [20,25]. SP-A and SP-D are collagen-containing (C-type) calcium-dependent lectins called collectins [113]. SP-D forms oligomers of 4–8 subunits. Each subunit is composed of three identical polypeptides of 43 kDa held together by disulfide bonds and non-covalent interactions at the *N*-terminal ends of the chains. Each polypeptide consists of a short *N*-terminal region, followed by a collagen-like sequence, a short α-helical sequence and the carbohydrate recognition domain. DMBT1^GP340^ binds to the carbohydrate recognition domain of SP-D through a calcium-dependent protein-protein interaction [20]. SP-A and SP-D are involved in a range of immune functions including viral neutralization, aggregation and killing of bacteria and fungi, and clearance of apoptotic and necrotic cells.

## 13. Role of DMBT1 in Innate Immunity

Due to their broad pathogen binding spectrum, DMBT1 and its mouse homolog CRP-ductin are considered pattern recognition receptors (PRRs) [101], an ancient germline encoded defense system against pathogen invasion in both plants and animals [114,115]. PRRs recognize conserved structures of the microbial cell that are vital for survival, so–called pathogen-associated molecular patterns (PAMPs). PAMPs include mannans and zymosan of the yeast cell wall, various bacterial cell-wall components, such as lipoteichoic acid (LTA) of Gram-positive bacteria or lipopolysaccharide (LPS) of Gram-negative bacteria, and bacterial DNA [116,117]. In the human body, PRRs are found on host–pathogen interacting surfaces and expressed by epithelial cells, macrophages, granulocytes and dendritic cells or as secretory products present in mucosal fluids [115,118]. These characteristics are also applicable to DMBT1. PRRs form a diverse group of proteins including mannose-binding lectin, collectins, CD14, Toll-like receptors and Nucleotide-binding oligomerization domain (Nod) proteins [70,115,119,120]. The SRCR proteins SR-AI and SR-AII, MARCO and Spα, also belong to this group.

## 14. Conclusions

Conclusively, DMBT1 plays a role in innate immunity by binding to a large panel of exogenous and endogenous ligands. SRCRP2 on the SRCR domains and sialic acids on the glycan chains have been identified as ligand binding sites, but DMBT1 may harbor many other potential ligand binding sites. These many ligand binding sites and its structural heterogeneity—both in the different tissues and between different individuals—are only two of the many surprises hidden in this molecule. Future research should focus on the precise role of DMBT1 in the innate immune system and the physiological consequences of its heterogeneity.

## Figures and Tables

**Table 1.** DMBT1 synonyms and orthologs in different organisms.

**Organism**	**Name**	**Function**
Human	DMBT1	Tumor suppression
	gp-340	Epithelial cell differentiation
	SAG	Innate immunity

Mouse	CRP-ductin	Mucosal defense
	Vomeroglandin	Epithelial differentiation
	Muclin	Pheromone perception
	apactin	Sorting receptor

Rabbit	Hensin	Terminal differentiation of kidney epithelial intercalated cells and embryonic stem cells

Rat	Ebnerin	Liver regeneration
	Pancrin	Taste perception

Pig	Porcine dmbt1	

Cow	Bovine gallbladder	Cholesterol-binding
	mucin	Gallstone formation

Rhesus monkey	H3	Hormone-responsive
		Endometrial regeneration in ovulation cycle

**Table 2.** Overview of glycan chains found on *DMBT1*.

**Oligosaccharide**	**Structure**	**Method**	**Source**	**Reference:**
core 1	Galβ1-3GalNAcα1-Ser/Thr			
sialylated-core 1	NeuAcα2-3 Galβ1-3GalNAcα1-Ser/Thr	LC-MS^2^	Tears/Saliva	[61,64]
disialylated-core 1	NeuAcα2-3Galβ1-3(NeuAcα2-6)GalNAcα1-Ser/Thr			
core 2	Galβ1-3(GlcNAcβ1-6)GalNAcα1-Ser/Thr	LC-MS^2^	Tears/Saliva	[61,64]
sialylated-core 2	NeuAcα2-3 Galβ1-3(GlcNAcβ1-6)GalNAcα1-Ser/Thr			

(Le-a)	Galβ1-4(Fucα1-3)GlcNAc	immunoblotting	Saliva	[13]
(Le-x)	Galβ1-3(Fucα1-4)GlcNAc	immunoblotting	Saliva	[13,22]
(Le-b)	Fucα1-2 Galβ1-4(Fucα1-3)GlcNAc	immunoblotting/LC-MS^2^	Saliva	[13,64]
(Le-y)	Fucα1-2Galβ1-3(Fucα1-4)GlcNAc	immunoblotting/LC-MS^2^	Saliva	[13,64]
H antigen	Fucα1-2 Galβ1-4GlcNAc	immunoblotting/LC-MS^2^	Saliva	[13,64]
A antigen	GalNAcα1-3(Fucα1-2)Galβ1-4GlcNAc	immunoblotting	Saliva	[13]
B antigen	Galα1-3(Fucα1-2)Galβ1-4GlcNAc	immunoblotting	Saliva	[13]
(Sialyl-Le^x)^	NeuA-α2-3Galβ1-3(Fucα1-4)GlcNAc	immunoblotting	saliva, not lung	[22]
Sialyl-Le^a^	NeuAcα2-3 Galβ1-4(Fucα1-3)GlcNAc	Immunoblotting/LC-MS^2^	Tears	[61]]
	NeuAcα2-3	lectin blotting	Saliva, not lung	[60]
	NeuAcα2-6	lectin blotting	lung, saliva	[60]
	SO_3_-6GlcNAc	Immunoblotting	Saliva	[63]

**Table 3.** Binding sites for DMBT1^SAG^ on various proteins.

**Protein**	**Sequence**	**SRCRP2 binding**	**Ref.**
*S.mutans* antigen I/II	PQLKTADLPAGRDETTSFVLV		[85]

*S.gordonii* antigen I/II	ELKTEALTAGRPKTTSFVLV		[86]
*S.mutans* antigen I/II	QLKTADLPAGRDETTSFVLV		

*S.gordonii* SspB 390-402	DYQAKLAAYQTEL DYQAKLAAYQKEL	+	[87]
*S.gordonii* SspB 390-T400K-402		+	

SsP(A4K-A11K)	DYQKKLAAYQKEL	+	[88]
Consensus SspA and B	DYQAKLAAYQAEL	+	

Bovine lactoferrin 480-492	SCAFDEFFSQSCA	+	[89,90]

Human lactoferrin	DCKFDEYFSQSCA		[91]

Human casein	LLNQELLNPTHQIYPVTQPLAPVHNPISV		[91]

HIV V3 region	CTRPNYNKRKR		[92]
